# Soft Interference Cancellation for Random Coding in Massive Gaussian Multiple-Access †

**DOI:** 10.3390/e23050539

**Published:** 2021-04-28

**Authors:** Ralf R. Müller

**Affiliations:** Institute for Digital Communications, Friedrich-Alexander Universität Erlangen-Nürnberg, 91058 Erlangen, Germany; ralf.r.mueller@fau.de

**Keywords:** multiple-access, successive cancellation, iterative decoding, finite blocklength, block error probability, random coding, AWGN, low-latency communications, spectral efficiency, non-othogonal multiple-access

## Abstract

In 2017, Polyanskiy showed that the trade-off between power and bandwidth efficiency for massive Gaussian random access is governed by two fundamentally different regimes: low power and high power. For both regimes, tight performance bounds were found by Zadik et al., in 2019. This work utilizes recent results on the exact block error probability of Gaussian random codes in additive white Gaussian noise to propose practical methods based on iterative soft decoding to closely approach these bounds. In the low power regime, this work finds that orthogonal random codes can be applied directly. In the high power regime, a more sophisticated effort is needed. This work shows that power-profile optimization by means of linear programming, as pioneered by Caire et al. in 2001, is a promising strategy to apply. The proposed combination of orthogonal random coding and iterative soft decoding even outperforms the existence bounds of Zadik et al. in the low power regime and is very close to the non-existence bounds for message lengths around 100 and above. Finally, the approach of power optimization by linear programming proposed for the high power regime is found to benefit from power imbalances due to fading which makes it even more attractive for typical mobile radio channels.

## 1. Introduction

Massive multiple-access is a key component of the upcoming internet-of-things. In contrast to classical settings, the number of devices typically exceeds the number of bits which an individual device aims to communicate. Therefore, it makes sense to consider different asymptotics for massive multiple-access: Keep the message length fixed, but let the number of devices grow over all bounds. This is in contrast to the classical setting in information theory where the message length becomes infinitely large, but the number of devices remains constant.

This new asymptotic setting was first discussed in [1] and further developed in [2] for static, non-faded channels. A key observation of [2] is that a new definition of error probability is appropriate: It is sufficient if most devices are able to decode their messages correctly. Thus, we refer to the per-device probability of error in the sequel, even if this is not stated explicitly.

A similar asymptotic setting, focusing on bit error probability and convolutional codes concatenated with random spreading, was first analyzed in [3], see also [4]. Qualitatively similar conclusions as in [2] were reported: The spectral efficiency grows without need for larger energy per bit up to some limit. Only beyond that limit, additional energy is required to further increase spectral efficiency. So, the behaviors fundamentally differ in the low and the high power regime.

The considered multiple-access setting is similar to sparse superposition codes on the single-user additive-white Gaussian noise (AWGN) channel which were found able to achieve channel capacity in [5]. For certain rates, however, iterative decoding methods show convergence issues. Similar to what is reported in [4], these issues can be overcome by power profile optimization [6,7].

The existence bounds found in [2] were improved in the subsequent work [8] which managed to very tightly quantify the tradeoff between spectral and power efficiency in the regime of high signal-to-noise ratio (SNR). For low SNR, the gap between the two bounds has remained significant. Furthermore, the bounds in [2,8] were obtained by non-constructive means, i.e., just as Shannon’s 1948 random coding argument, they do not hint towards any algorithm that is capable to achieve them closely, in practice. Therefore, this work intends to pursue the following three aims:

(1) Improve the theoretical bounds in [8].
(2) Propose coding and decoding schemes with polynomial complexity that closely approach the performances promised by these bounds.
(3) Investigate in which way these results for static channels carry over to fading channels.

In order to achieve these goals, the following methods are combined:

(A) Iterative soft cancellation of interference, i.e., only an attenuated version of the estimated interference is subtracted from the receive signal to reduce the potentially harmful effect of error propagation [9,10].
(B) Treating residual interference as independent additive white Gaussian noise.
(C) Recent calculations of the exact ensemble-averaged block-error probability of independent identically distributed (iid) Gaussian random codes in [11].
(D) Orthogonal constellations as efficient block codes with low rate.
(E) Finding the fixed-point of the iterations by tracking the evolution of the multiuser efficiency of all devices as pioneered in [12].
(F) Power profile optimization by linear programming as proposed in [3,4] to cope with the high power regime.

In order to achieve the three aims stated above, the paper is organized as follows: In Section 2, the system model and iterative soft interference cancellation, i.e., Method A, is introduced for an arbitrary number of devices. Section 3 is concerned with the analysis of the proposed soft interference cancellation in the limit of infinitely many devices. First, Section 3.1 finds the infinite device limit for the ensemble averaged posterior block error probability of Gaussian random coding at fixed message length for a given amount of residual interference combining Methods B and C. Then, this block error probability is utilized in Section 3.2 to find approximate upper and lower bounds on the amount of residual interference after soft cancellation and track their evolution combining Methods D and E. Finally, Section 3.3 utilizes Method F to improve the convergence of the iterations and the tightness of the bounds in the high power regime and completes the large-system analysis. Section 4 addresses the influence of fading and shows that it is actually helpful in the high power regime. Section 5 discusses numerical results and Section 6 outlines conclusions and implications.

## 2. System Model

Let there be *M* devices with codewords $c_{1},\ldots ,c_{M}$ that want to communicate over the Gaussian multiple-access channel

(1) $$r=\sum_{m=1}^{M}c_{m}+n$$

with AWGN n of unit covariance, i.e., E$nn^{†}=I$. Every device wants to transmit *K* information bits and encodes them into the codeword $c_{m}\in R^{MN}$ for some *N* such that MN∈Z. The codeword $c_{m}$ is chosen from the set $C_{m}$ of $2^{K}$ jointly iid Gaussian codewords by a bijective mapping to the information bits of device *m*. The codebooks of different devices are chosen statistically independent from each other.

Let the total set of all devices be decomposed into a finite number of disjoint groups $G_{1},\ldots ,G_{J}$. Within group $G_{j}$, the power of every device is given by $P_{j}/M$, i.e., E$c_{m}c_{m}^{†}=P_{j}I/M$. The powers of the devices are equal within each group, but differ from group to group. The fraction of devices in group $G_{j}$ is denoted by $\alpha_{j}=\left|G_{j}\right|/M$. The aggregate power of all devices is denoted by

(2) $$P=\sum_{j=1}^{J}\alpha_{j}P_{j}.$$

The devices are solely grouped to improve the convergence of successive cancellation by means of power control, cf. Section 3.3; see [4,6,7] for detailed reasons on this device grouping. All devices transmit independently from any other device in the same or a different group.

Let

(3) $$R=\frac{K}{N}$$

denote the aggregate rate of all devices. It is sometimes referred to as spectral efficiency. The meaning of the variable *N* is not intuitively clear. In fact, it is a free parameter for system design. In the single device case (M=1), it is the blocklength of the code. In [8], its reciprocal 1/N is called *user density*.

Let all devices use parallel successive decoding in an iterative manner. That means all devices are decoded in parallel resulting in estimated codewords ${\hat{c}}_{m}$. Then, the interference is estimated for all devices and cancelled from the received signal, before all devices are decoded again with (hopefully) lower error probability than initially. For any device *m*, the new estimate at iteration i+1 is formed from the estimate at iteration *i* by

(4) $${\hat{c}}_{m}^{\left(i+1\right)}=f\left(r-\sqrt{s^{i)}}\sum_{j=1}^{J}\sqrt{s_{j}^{\left(i\right)}}\sum_{m^{\prime }\in G_{j}\setminus \left\{m\right\}}q_{m^{\prime }}^{\left(i\right)}{\hat{c}}_{m^{\prime }}^{\left(i\right)}\right)$$

for some soft-cancellation coefficients $q_{m}^{\left(i\right)}$ and renormalization factors $s^{\left(i\right)}$ and $s_{j}^{\left(i\right)}$ to be specified later on, as well as some decoding function f(·). This process is repeated until a steady state is reached.

During iterations, the estimates of the codewords of the devices become correlated. Thus, it is not ideal to estimate the aggregate interference by directly summing the interference contributions of all interfering devices. In the sequel, we propose a simple low cost countermeasure that, in Section 5, turns out to work, though it also leaves room for further improvements.

In order to cancel interference, an estimate for the interfering signal due to group $G_{j}$ is calculated for all groups. The estimate is formed by

(5) $${\hat{ı}}_{j}={\sqrt{s}}_{j}\sum_{m\in G_{j}}q_{m}{\hat{c}}_{m}$$

with $s_{j}$ being a renormalization factor that will be discussed in the sequel.

If all interference estimates $q_{m}{\hat{c}}_{m}$ in (5) were uncorrelated, the total interference power would be given by

(6) $$E||{\hat{ı}}_{j}{\left|\right|}^{2}=\frac{s_{j}P_{j}}{M}\sum_{m\in G_{j}}q_{m}^{2},$$

since $E||{\hat{c}}_{m}{\left|\right|}^{2}=P_{j}/M$ for devices in group $G_{j}$. Since the interference estimates $q_{m}{\hat{c}}_{m}$ are not uncorrelated, the estimated interference is typically larger. This overestimation leads to a too aggressive interference cancellation policy which is prone to error propagation. To avoid such harm, we set

(7) $$s_{j}=\frac{\frac{P_{j}}{M}\sum_{m\in G_{j}}q_{m}^{2}}{{\left|\left|\sum_{m\in G_{j}}q_{m}{\hat{c}}_{m}\right|\right|}^{2}}.$$

An additional minor improvement is achieved, if the re-normalization is repeated among device groups. The total estimate of interference is, thus, formed as

(8) $$\hat{ı}=\sqrt{s}\sum_{j=1}^{J}{\hat{ı}}_{j}$$

with

(9) $$s=\frac{\sum_{j=1}^{J}\frac{P_{j}}{M}\sum_{m\in G_{j}}q_{m}^{2}}{{\left|\left|\sum_{j=1}^{J}{\hat{ı}}_{j}\right|\right|}^{2}}.$$

These two re-normalizations of the interference estimate strongly improve the block error rate simulated in Section 5.

## 3. Large-System Analysis

The signals of all devices initially fully interfere with each other. After some iterations, only a certain fraction $v_{j}$ of the interference power, which group $G_{j}$ had initially contributed, remains due to partially successful cancellation of interference. At this point, the aggregate power of interference and noise is given as

(10) $$I=1+\sum_{j=1}^{J}\alpha_{j}v_{j}P_{j}$$

in the large device limit M→∞, as the power of the device of interest vanishes.

### 3.1. Asymptotic Block Error Probability

Given a certain fraction of remaining interference, we want to calculate the posterior (conditional) block error probability of the decoder averaged over the random code ensemble in the large device limit M→∞. We will need this block error probability in Section 3.2 to find the fixed-point of the iterative cancellation process.

We start with the unconditional block error probability which is calculated in Appendix A utilizing recent results in [11].

**Theorem** **1.** *Given the Gaussian multiple-access channel defined in (1) and residual interference treated as AWGN, the unconditional block error probability of any device in group $G_{j}$ averaged over the random code ensemble of this same device converges almost surely to*

(11) $$\begin{array}{cc} p_{j} & =1-\int_{R}Q{\left(x-\sqrt{\eta NP_{j}}\right)}^{2^{K}-1}Dx \end{array}$$

*for M→∞ with $Dx:=e^{-x^{2}/2}/\sqrt{2\pi }dx$ denoting the Gaussian measure and*

(12) $$\eta =\frac{1}{1+\sum_{j=1}^{J}\alpha_{j}v_{j}P_{j}}$$

*denoting the multiuser efficiency [13].*

The unconditional block error probability (11) is the symbol error probability of a $2^{K}$-dimensional orthogonal constellation in AWGN and can already be found in [14], see also ([15] 5.2-21). All codewords of all devices are asymptotically pairwise orthogonal to each other in the large device limit. This is a special case of a stronger result in [16]:

**Theorem** **2.** *Let there be n iid zero-mean Gaussian random vectors in βn dimensions with 0<β<∞. Let α be the cosine of the smallest angle between any pair of them. Then, $\alpha \sqrt{n/\ln n}$ converges almost surely to 2, as n→∞.*

Note, however, that asymptotic pairwise orthogonality does not imply that codewords do not interfere with each other. Even if the interference due to the codeword of an individual device vanishes, the aggregate interference of infinitely many devices may be strictly positive.

The asymptotic orthogonality allows us to calculate some posterior block error probabilities in the large device limit $MN\gg 2^{K}$. Consider an alternative Cartesian coordinate system in $2^{K}$ dimensions that results from the original coordinate system by the following 2-step procedure:

1. an orthonormal transformation such that ${\tilde{c}}_{k}$, denoting the kth codeword of the codebook of the device of interest, is a positive multiple of the kth unit vector, for all $1\leq k\leq 2^{K}$.
2. the removal of all coordinates with indices greater than $2^{K}$.

The orthonormal transformation ensures that the statistical properties of all signals are preserved. Treating residual interference as AWGN, the dropped coordinates do not contain useful information about the data of the device of interest.

Let the $\tilde{r}=\left[{\tilde{r}}_{1},\ldots ,{\tilde{r}}_{2^{K}}\right]$ denote the received vector in the new coordinate system. The tildes serve to distinguish the original coordinate system in MN dimensions from this newly introduced one in $2^{K}$ dimensions. Assume that codeword ${\tilde{c}}_{1}$ has been sent and define

(13) $$\begin{array}{c} {\tilde{r}}_{k:}=\max\left\{{\tilde{r}}_{k},{\tilde{r}}_{k+1},\ldots ,{\tilde{r}}_{2^{K}}\right\}. \end{array}$$

Note that ${\tilde{r}}_{1}$ and ${\tilde{r}}_{2:}$ are statistically independent and ${\tilde{r}}_{1:}=\max\left\{{\tilde{r}}_{1},{\tilde{r}}_{2:}\right\}$. With these definitions, a decoding error occurs, if ${\tilde{r}}_{2:}>{\tilde{r}}_{1}$. Conditioning on the largest component of the receive word ${\tilde{r}}_{1:}$, we get the posterior block error probability

(14) $$\begin{array}{cc} p_{j|{\tilde{r}}_{1:}} & =\mathrm{Pr}({\tilde{r}}_{1}<{\tilde{r}}_{2:}|{\tilde{r}}_{1:}) \end{array}$$

(15) $$\begin{array}{cc} \; & =\frac{\int_{R}P_{{\tilde{r}}_{1}}\left({\tilde{r}}_{2:}\right)\;p_{{\tilde{r}}_{2:}}\;\left({\tilde{r}}_{2:}\right)\delta \left({\tilde{r}}_{2:}-{\tilde{r}}_{1:}\right)\;d{\tilde{r}}_{2:}}{p_{{\tilde{r}}_{1:}}\;\left({\tilde{r}}_{1:}\right)}=\frac{P_{{\tilde{r}}_{1}}\left({\tilde{r}}_{1:}\right)\;p_{{\tilde{r}}_{2:}}\;\left({\tilde{r}}_{1:}\right)}{p_{{\tilde{r}}_{1:}}\;\left({\tilde{r}}_{1:}\right)} \end{array}$$

utilizing Bayes’ law with $P_{a}\left(\cdot \right)$ and $p_{a}\left(\cdot \right)$ denoting cumulative distribution function and probability density function of *a*, respectively. The Dirac function δ(·) occurs, since ${\tilde{r}}_{1}<{\tilde{r}}_{2:}$ implies ${\tilde{r}}_{2:}={\tilde{r}}_{1:}$. Furthermore, exchanging random variables ${\tilde{r}}_{1}$ and ${\tilde{r}}_{2:}$ in (14) gives the probability of the complementary event. Thus, we have

(16) $$p_{{\tilde{r}}_{1:}}\;\left({\tilde{r}}_{1:}\right)=P_{{\tilde{r}}_{2:}}\left({\tilde{r}}_{1:}\right)\;p_{{\tilde{r}}_{1}}\;\left({\tilde{r}}_{1:}\right)+P_{{\tilde{r}}_{1}}\;\left({\tilde{r}}_{1:}\right)\;p_{{\tilde{r}}_{2:}}\;\left({\tilde{r}}_{1:}\right).$$

which leads to

(17) $$\begin{array}{cc} p_{j|{\tilde{r}}_{1:}} & =\frac{1}{1+\frac{P_{{\tilde{r}}_{2:}}\;\left({\tilde{r}}_{1:}\right)\;p_{{\tilde{r}}_{1}}\;\left({\tilde{r}}_{1:}\right)}{P_{{\tilde{r}}_{1}}\;\left({\tilde{r}}_{1:}\right)\;p_{{\tilde{r}}_{2:}}\;\left({\tilde{r}}_{1:}\right)}}=\frac{1}{1+F\left({\tilde{r}}_{1:}/\sqrt{I}\right)} \end{array}$$

with implicit definition of F(·).

Since ${\tilde{r}}_{1}$ is a Gaussian random variable with mean $\sqrt{NP_{j}}$ and variance *I*, we have

(18) $$\begin{array}{cc} P_{{\tilde{r}}_{1}}\left(x\right) & =Q\left(\left(\sqrt{NP_{j}}-x\right)/\sqrt{I}\right). \end{array}$$

Furthermore, ${\tilde{r}}_{2},\ldots ,{\tilde{r}}_{2^{K}}$ are Gaussian random variables with zero mean and variance *I*. Thus, we have

(19) $$\begin{array}{cc} P_{{\tilde{r}}_{2:}}\left(x\right) & =Q{\left(-x/\sqrt{I}\right)}^{2^{K}-1}. \end{array}$$

With (18) and (19) and their derivatives, the posterior block error probability (17) can be evaluated for any observation ${\tilde{r}}_{1:}$. In particular, we find

(20) $$F\left(x\right)=\frac{Q\left(-x\right)e^{-\frac{1}{2}{\left(x-\sqrt{\eta NP_{j}}\right)}^{2}}}{Q\left(-x+\sqrt{\eta NP_{j}}\right)e^{-\frac{x^{2}}{2}}\left(2^{K}-1\right)}$$

where the interference power *I* was substituted by the multiuser efficiency η via (10) and (12).

The closed form expressions (14) and (20) will be used in the next subsection to track the evolution of residual interference during iterations.

### 3.2. Evolution of Residual Interference

In order to track the block error probability during iterations, we need to connect the fraction of remaining interference $v_{j}$ to the error probability at the previous iteration. This subsection serves exactly that purpose.

The remaining interference is determined by the way potential interference is cancelled. There are various ways of performing soft interference cancellation. Irrespective of the precise algorithm, the dynamics of the iterations can be studied by tracking the multiuser efficiency, as proposed in [12]. The advantage of tracking multiuser efficiency in comparison to, e.g., using extrinsic information transfer charts (see [17] for details), is the fact that the multiuser efficiency of all devices is equal in the large system limit ([12] Proposition 2). So only a single parameter needs to be tracked.

With (12), we have

(21) $$\begin{array}{cc} \eta^{\left(i\right)} & =\frac{1}{1+\sum_{j=1}^{J}\alpha_{j}v_{j}^{\left(i\right)}P_{j}} \end{array}$$

with $\eta^{\left(i\right)}$ and $v_{j}^{\left(i\right)}$ denoting the multiuser efficiency and the remaining fraction of interference in group $G_{j}$, both at iteration *i*. The goal of this section is to characterize the mapping

(22) $$\begin{array}{cc} \eta^{\left(i\right)} & \mapsto \left[v_{1}^{\left(i+1\right)},\ldots ,v_{J}^{\left(i+1\right)}\right] \end{array}$$

in order to track the evolution of the multiuser efficiency.

During iterations, the interference may become correlated to the true data. This is a severe issue, since decision rules based on Euclidean distance, as used in this work, require the statistical independence between data and interference. As found in the very related context of sparse superposition coding [7], such correlations do indeed occur. This problem is often addressed by means of approximate message passing [18,19] and its various recent improvements [20,21,22] via cancellation of the Onsager reaction terms; see [23] for details. Due to the multidimensional nature of the codebook, approximate message passing is anything but straightforward to apply to the problem at hand and is left for future work.

Correlations between data and noise are not as severe as for scalar interference cancellation with antipodal data due to the following property of the random code construction: The codewords of all devices are chosen statistically independent. Thus, they are orthogonal in the large device limit. This means that a wrong decision in iteration *i*, by means of erroneous cancellation, does not lead to an additional interference in iteration i+1 that points into the same direction as the true signal, as it would be the case for, e.g., binary antipodal constellations. In contrast, it creates additional interference that is orthogonal to the true data. For the sake of analytical tractability, we assume that the correlation of noise and data during iterations can be neglected. This implies that $s_{j}=s=1,\forall j$. We have to keep in mind that the results, obtained under this assumption, are not exact, but an approximation.

For the calculation of error probability, we rely on self-ergodicity. Self ergodicity means that, in an infinite population of independent devices, the relative frequency of decoding errors matches its statistical distribution. Thus, the instantaneous interference power after interference cancellation based on potentially erroneous decoding also equals its statistical expectation.

If we have received word r and decided for a codeword ${\hat{c}}_{m}$, this decision is correct with probability $1-p_{j|r}$ for all $m\in G_{j}$. Paying tribute to potentially wrong decisions, we do not fully subtract the codeword ${\hat{c}}_{m}$ from the received word r, but only subtract $q_{m}\;{\hat{c}}_{m}$ with some soft-cancellation factor $0\leq q_{m}\leq 1$, cf. (4). In the large system limit, the error probabilities within each group are identical due to self-ergodicity and so are the soft-cancellation factors. So, we can set $q_{m}=q_{j|r}$. After soft cancellation, the remaining interference power due to any device in group $G_{j}$ is

(23) $$\left[{\left(1-q_{j|r}\right)}^{2}\left(1-p_{j|r}\right)+\left(1+q_{j|r}^{2}\right)p_{j|r}\right]\frac{P_{j}}{M}$$

on average. Note again that all codewords are orthogonal. In case of erroneous cancellation, the interference does not add in amplitude, but in power. Direct optimization of (23) leads to the soft-cancellation rule

(24) $$q_{j|r}=1-p_{j|r}.$$

Together with (23), the fraction of remaining interference becomes

(25) $$v_{j}=1-\underset{r}{E}{\left(1-p_{j|r}\right)}^{2}.$$

In order to implement (24), we need to know $p_{j|r}$, the error probability within device group $G_{j}$ given the receive word r.

Since we do not know how to calculate $p_{j|r}$, we will use upper and lower bounds on the fraction of remaining interference. For the upper bound, we base our soft-cancellation on $p_{j|{\tilde{r}}_{1:}}$ instead of $p_{j|r}$. This yields

(26) $$\begin{array}{cc} v_{j}<v_{j}^{u} & =1-\int_{R}\frac{Q{\left(-\frac{x}{\sqrt{I}}\right)}^{2^{K}-1}e^{-\frac{{\left(x-\sqrt{NP_{j}}\right)}^{2}}{2I}}}{\left[1+1/F\left(x\right)\right]\sqrt{2\pi I}}\;dx \end{array}$$

(27) $$\begin{array}{cc} \; & =1-\int_{R}\frac{Q{\left(x-\sqrt{\eta NP_{j}}\right)}^{2^{K}-1}}{1+1/F\left(\sqrt{\eta NP_{j}}-x\right)}\;Dx. \end{array}$$

For the lower bound, we assume perfect knowledge of whether a decision is correct or not. This implies

(28) $$v_{j}>v_{j}^{l}=p_{j}.$$

In the sequel, we will refer to these bounds when addressing the performance of decision-directed soft-cancellation. A comparison of (27) and (11) shows that the two bounds only differ by the denominator in (27).

### 3.3. Improving Convergence

Irregularity aids the convergence of iterative systems. This phenomenon is well studied, e.g., in the context of low-density parity check codes [24]. It has also been observed for iterative multiuser decoding in [3]. There are various ways to introduce irregularity into iterative multiuser decoding. In the sequel, we will address power imbalances among devices.

While for low rates, equal power levels for all devices turn out optimal, this does not hold if the rate exceeds some finite threshold. This effect was first observed in [3] and also reported for sparse superposition codes in [6,7]. In the sequel, we apply the ideas of power optimization laid out in [3] to Gaussian random coding assuming an infinite number of devices.

Power optimization can be performed by linear programming. This is possible, as the multiuser efficiency is identical for all device groups. Its evolution during iterations can be tracked by the dynamical system defined in (21) and (22). The mappings from the multiuser efficiency to the fractions of remaining interference depend on the particular way, interference cancellation is implemented. For the upper and lower bounds considered in this paper, they can be found in (27) and (28) via (11).

In order for iterations to converge, we need to ensure that the multiuser efficiency at the next iteration exceeds the current multiuser efficiency by at least an arbitrarily small margin ϵ>0. This can be ensured by the linear program

(29) $$\left\{\begin{array}{cc} \min_{\alpha_{1},\ldots ,\alpha_{J}} & \sum_{j=1}^{J}\alpha_{j}P_{j}\\ \mathrm{subject}\;\mathrm{to} & \alpha_{j}\geq 0\;\forall j\\ \; & \sum_{j=1}^{J}\alpha_{j}P_{j}v_{j}\left(\eta \right)<\frac{1}{\eta +\epsilon }-1\;\forall \eta \in E\\ \; & \sum_{j=1}^{J}\alpha_{j}=1 \end{array}\right..$$

for appropriately chosen interval E⊂[0;1] and margin ϵ. The upper end of the interval and the margin are design parameters of the multiuser system. The smaller the margin is, the more iterations are needed. The choice of E, however, is not trivial at all. The lower end can be chosen arbitrarily close to 0, but eventually also somewhat larger to speed up the linear program as long as it does not exceed the multiuser efficiency before the first iteration. The choice of the upper end determines the final error probability by means of a strictly monotonous function (more remaining interference implies higher error probability). It is typically close to one.

The powers $P_{j}$ are quantized versions of the optimal distribution of powers. The larger the number of groups *J*, the better is the approximation to the optimal distribution. This indirect way of power optimization is chosen, as the function $v_{j}\left(\eta \right)$ depends in a non-convex way on the powers of the devices, see (27), but does not involve the group size. In this way, the optimization can be efficiently solved by linear programming and takes only few seconds on a desktop computer.

## 4. The Near-Far Gain

In practice, receive powers of devices will vary anyway due to different propagation conditions among devices. This can be utilized to reduce the average transmit energy per bit following the ideas of [25], see also ([26] Chapter 5) and [27]. In [28], the term near-far gain was coined to refer to this convenient property of wireless systems in order to emphasize that the near-far effect does not cause problems, but is actually beneficial, if the system is designed in the right way. A similar concept was popularized more recently under the generic term non-orthogonal multiple-access (NOMA) [29]. In context of the current work, one simply needs to slightly adjust the objective function of (29).

The origin of the near-far gain is sometimes obscured in recent papers on NOMA. In fact, the near-far gain is difficult to understand intuitively, if one is too focussed on a direct boost in data rate. Information theory, however, establishes a fundamental duality between data rate and energy per bit. If our aim is to minimize the energy per bit for a given target data rate instead, the near-far gain is very intuitive, as explained in the sequel.

For iterative decoding and/or successive cancellation to work close to capacity limits, irregularity is required, in general. This irregularity can be provided by the system design at some price, e.g., protecting some data symbols by more parity-checks than others. This comes at the expense of more redundancy and, thus, reduced data rate. In successive cancellation, the equivalent is larger transmit power. Here the price is paid in dual currency: in the energy per bit.

Near-far situations provide irregularity for free. It takes the form of receive power imbalances. These natural receive power imbalances are not exactly distributed as they are supposed to be. Adjustment is needed. However, it takes less effort to adjust from already imbalanced receive powers than starting from the worst case: equal received powers. The reduced adjustment effort is the near-far gain measured in reduced transmitted energy-per bit. It may be quantified running the linear program (33) once with unit weights and once with weights provided by natural attenuation, then comparing the two total powers (2). Standard methods can be applied for currency conversion into bits/s/Hz.

The near-far gain is not restricted to path loss alone. Long-term fading typically exhibits dynamics slow enough to be utilized in the same or a similar way. For some systems, even short-term fading may be utilized. These details have been extensively discussed in the recent NOMA literature, cf. [29] for a survey.

## 5. Numerical Results

Numerical results can be difficult to obtain. If the number of bits per device exceeds values around 35, the exponent $2^{K}-1$ in various equations becomes numerically unstable to evaluate, as the basis is very close to one. This can be circumvented as follows:

(30) $$Q{\left(x\right)}^{a}=e^{a\ln(1-Q(-x))}=\prod_{i=1}^{\infty }e^{-aQ{\left(-x\right)}^{i}/i}$$

For sufficiently large *a*, all factors for i>1 are so close to one that they can be ignored; see [11] for details. Furthermore, the Gaussian integration can be tedious. We recommend Gauss–Hermite quadrature with several hundred terms (we use 300 in this work).

### 5.1. Equal Path Loss for All Devices

**Figure 1:**
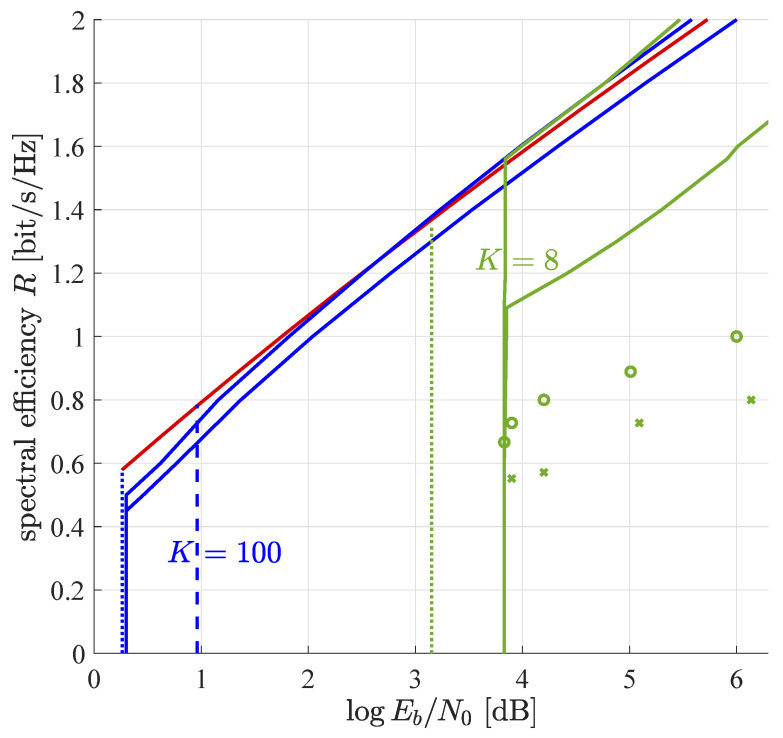
Spectral efficiency vs. rate-compensated SNR for per device block error rate $10^{-3}$. The solid lines refer to our inner and outer bounds introduced in Section 3.2. The dashed and dotted lines refer to the best inner and outer bounds of [8]. The two indistinguishable red lines are given by setting $w_{\ell }=1$ in (36) for K=100 and K=8. Points marked by circles and crosses refer to simulations with 256 and 32 devices in the largest group, respectively.

Figure 1 shows the trade-off between spectral efficiency and power efficiency for block error rate $10^{-3}$, the power distribution optimized among devices with parameter $\epsilon =10^{-3}$, and equal message lengths for all devices. In order to find the upper end of the interval E, the method of interval nesting was applied and typical values for target error probability $10^{-3}$ were found to range from 0.95 to 0.99 depending on the signal-to-noise ratio. Figure 1 shows that there are two different paradigms: the equal power regime and the distributed power regime.

#### 5.1.1. Equal Power Regime

In the equal power regime, all devices transmit at the same power. In this regime, our outer and inner bounds on power efficiency coincide and spectral efficiency is independent of power efficiency. Iterations proceed until the multiuser efficiency approaches unity closely and nearly all interference is removed. Thus, the error probability relates to $E_{b}/N_{0}$ approximately as

(31) $$P_{e}=1-\int_{R}Q{\left(x-\sqrt{2K\frac{E_{b}}{N_{0}}}\right)}^{2^{K}-1}Dx.$$

In this regime, the error probability is determined by the minimum required $E_{b}/N_{0}$ and the number of information bits per device, i.e., *K*.

#### 5.1.2. Distributed Power Regime

In the distributed power regime, the sizes of the device groups are optimized by the linear program (29). Within each group, the power per device is the same, but it differs from group to group. In order to reduce granularity effects of the discretization of the power distribution, the linear program is run with more than hundred power groups. However, the linear program returns most of them empty (with zero devices). This indicates that the optimum number of groups is finite. Numerical results of the optimum power grouping are shown in Table 1 and Table 2 for K=8 and K=100, respectively. The larger spectral efficiency and SNR, the larger is the optimal number of groups. For the minimal SNR, all devices are in the same group. Any larger SNR has its own individually optimal power distribution. All these observations are in line with the results on power optimization in iterative decoding of convolutionally encoded code division-multiple access reported in [4]. The optimal power distributions in this reference are qualitatively very similar to the ones found in this work.

Intuitively, the power grouping solves the convergence issues in the following way: There is a certain maximum number of equal power devices that can be handled by successive interference cancellation. If the number of devices exceeds a certain threshhold, decoding drowns in interference and iterations do not converge to a steady state with few, but one with many errors. If we want to go beyond that threshhold, we need to give the additional devices such a large power that they can be decoded almost error-free in the presence of the lower power devices. Then, they can be cancelled perfectly, they do not interfere any longer and the iterative decoding of the low power users can start. If the number of devices increases further, we will, at some point, begin to need a third, forth, fifth group, and so on.

For large values of spectral efficiency, the outer bound of [8] (red line in Figure 1) becomes tighter than our outer bound which is based on the genie-added lower bound on the remaining interference (28). For K=100, inner bound and best outer bound differ by about a quarter of a decibel, while for K=8, they differ by approximately 1.5 dB.

#### 5.1.3. Finite Number of Devices

Simulations for a finite number of devices utilizing double re-normalization are shown as circles and crosses in Figure 1. For all simulation points 25,000 symbols are transmitted and up to 30 iterations are performed. For the fives simulation points around $\log E_{b}/N_{0}=4$ dB a single group of devices was used with M=256 and M=32 for the circles and the crosses, respectively. Performance strongly increases with the number of devices, as the codewords become more and more orthogonal and the variations of the multiuser efficiency around its large-system limit become smaller and smaller. Unfortunately, simulations with larger number of devices were not feasible on the author’s computer due to lack of memory.

For the simulation points at 5 dB and above, the power profile is optimized by try and error, as the linear program (29) cannot be utilized for finite *M*. To keep the size of the search space reasonable, only J=2 groups are considered. In all cases, the largest group of devices contains 256 and 32 devices, respectively, that operate with minimum power to achieve the target block error rate of $10^{-3}$. At average $\log E_{b}/N_{0}\approx 5$ dB, a second group with 64, respectively 8, devices of larger power is added. This increases the total number of devices to 320, respectively 40. Due to the devices with higher power, the average $E_{b}/N_{0}$ raises. At the same time, the parameter *N* can be reduced, such that the spectral efficiency increases, as well. At average $\log E_{b}/N_{0}\approx 6$ dB, the second device group is chosen twice as large as for $\log E_{b}/N_{0}\approx 5$ dB. Although the simulation results fall quantitatively behind the theoretical predictions for M→∞, they show the same qualitative behavior as the proposed theory. Recent subsequent works [30,31] show that simulations based on approximate message passing instead of basic soft interference cancellation perform well between the asymptotic bounds proposed in this paper.

#### 5.1.4. Minimum Signal-to-Noise Ratio

**Figure 2:**
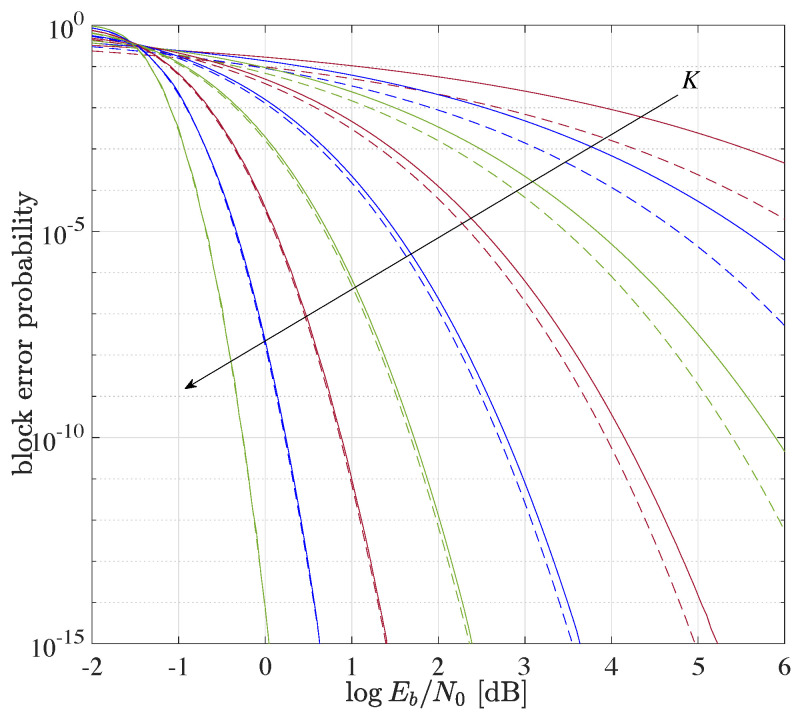
Block error probability at minimum required $E_{b}/N_{0}$ for various message lengths K=4,8,…,512,1024 (following arrow). Solid and dashed lines refer to (31) and (32), resp.

The block error probability at the minimum possible $E_{b}/N_{0}$ is shown in Figure 2 for various message lengths *K*. The solid and dashed lines refer to (31) and the lower bound [32]

(32) $$P_{e}>1-Q\left(Q^{-1}\left(2^{-K}\right)-\sqrt{2K\frac{E_{b}}{N_{0}}}\right)$$

respectively. While the lower bound is tight for long messages, it is considerable loser for short messages, where it may deviate from the exact result by even several orders of magnitude. The looseness for K=8 can also be observed in Figure 1.

### 5.2. Discretized Path Loss Model

Path loss is commonly modeled by a continuous statistical distribution. The linear program (29), however, can only handle a finite number of different received power levels. Therefore, we use a simple discretized model, in the sequel.

Let there be only *L* different fading weights ${\sqrt{w}}_{1},\ldots ,{\sqrt{w}}_{L}$. Partition each of the *J* device groups into *L* subgroups with the ℓth subgroup experiencing fading gain ${\sqrt{w}}_{\ell }$ and $\alpha_{j\ell }$ denoting the fraction of devices in the ℓth subgroup of group $G_{j}$. We modify the linear programm (29) to read

(33) $$\left\{\begin{array}{cc} \min_{\alpha_{j\ell },\forall j,\ell } & \sum_{j=1}^{J}\sum_{\ell =1}^{L}\alpha_{j\ell }w_{\ell }P_{j}\\ s.t. & \alpha_{j\ell }\geq 0\;\forall j,\ell \\ \; & \sum_{j=1}^{J}\sum_{\ell =1}^{L}\alpha_{j\ell }w_{\ell }P_{j}v_{j}\left(w_{\ell }\eta \right)<\frac{1}{\eta +\epsilon }-1\;\forall \eta \in E\\ \; & \sum_{j=1}^{J}\alpha_{j\ell }=\mathrm{Pr}\left(w_{\ell }\right)\;\forall \ell \end{array}\right.$$

where we introduced additional constraints to prevent the linear program from changing the distribution of the fading gains.

Considering a linear path loss model and free space propagation (which gives similar results as a circular path loss model with attenuation exponent 4), we set the fading weights to

(34) $${\sqrt{w}}_{\ell }=\frac{1}{\ell }$$

and denote the average fading gain by

(35) $$\mu =\frac{1}{L}\sum_{\ell =1}^{L}w_{\ell }.$$

We redo the numerics of Figure 1 under otherwise identical conditions. However, we measure power efficiency in transmitted energy per bit normalized to the average fading gain, i.e., $E_{b}/\left(\mu N_{0}\right)$, which obeys the upper bound ([26] Equation (5.24))

(36) $$\frac{E_{b}}{\mu N_{0}}\geq \frac{1}{2R}\sum_{\ell =1}^{L}w_{\ell }\left[4^{aR\ell /L}-4^{aR(\ell -1)/L}\right].$$

**Figure 3:**
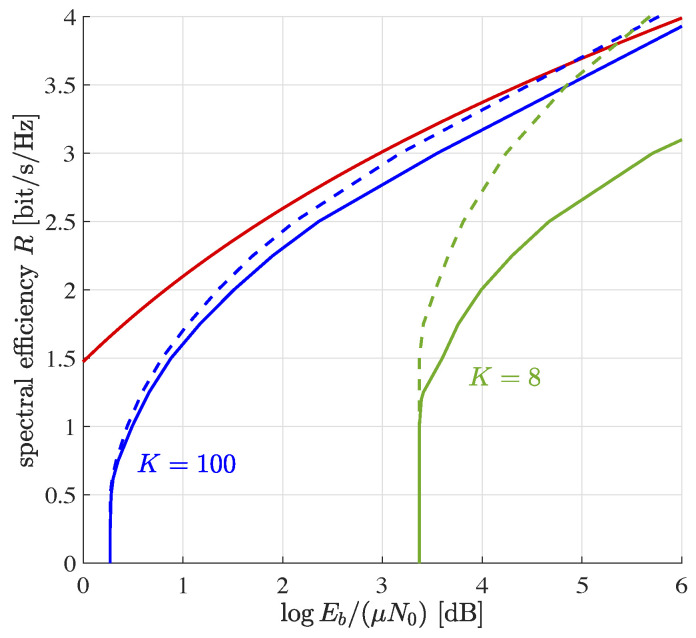
Spectral efficiency vs. rate-compensated transmit signal-to-noise ratio for per device block error rate $10^{-3}$. The two indistinguishable red lines are the outer bounds (36) for K=100 and K=8. All curves for L=10. The other lines refer to the inner and outer bounds introduced in Section 3.2.

Here,

(37) $$a=1-P_{e}-H_{2}\left(P_{e}\right)/K.$$

is a correction factor accounting for finite blocklength; see [8] for details. Numerical results are shown in Figure 3. In comparison to Figure 1, there is a smoother transition from the equal to the distributed power regime. The gap between our two bounds has widened.

The equal power regime has moved towards lower values of $E_{b}/\left(\mu N_{0}\right)$. The effect is particularly pronounced for short message lengths, cf. K=8. This happens, as there is no side constraint enforcing fairness among devices: While the overall block error probability is still $10^{-3}$, devices in bad channel conditions experience larger error probability. Devices in good channel conditions compensate for that. For devices in good channel conditions, low error probability is very cheap in terms of transmit power. As a result, this overcompensates for the excess power required by devices in bad channel conditions.

## 6. Conclusions

Random codes perform well for very massive multiple-access even if devices have short messages. They can be iteratively decoded by soft-cancellation of interference, but may required power optimization to create enough irregularity to allow iterations to converge.

In the large device limit, orthogonal constellations in $2^{8}$ dimensions carrying 8 information bits are hardly more than 1.5 dB behind random codes of infinite length, if spectral efficiency is larger than 1.1 bits/s/Hz. This gap is the larger, the smaller is the number of devices. Further research into iterative algorithms for soft cancellation, e.g., utilizing ideas of approximate message passing, may turn out helpful.

For high spectral efficiency, devices should be received at unequal power levels. This is beneficial in practice, as wireless propagation conditions unavoidably create such power imbalances. 

## Figures and Tables

**Table 1:** Optimal power profile for the approximate upper bound, K=8 information bits per device, and per device block error rate $10^{-3}$.

R=1	100%			average
$\log\frac{E_{b}}{N_{0}}$	3.82 dB			3.82 dB
R=1.2	81.6%	18.4%		average
$\log\frac{E_{b}}{N_{0}}$	3.76 dB	6.53 dB		4.42 dB
R=1.4	69.0%	31.0%		average
$\log\frac{E_{b}}{N_{0}}$	3.67 dB	7.56 dB		5.28 dB
R=1.6	63.3%	36.7%		average
$\log\frac{E_{b}}{N_{0}}$	3.64 dB	8.38 dB		6.01 dB
R=1.8	58.4%	41.6%		average
$\log\frac{E_{b}}{N_{0}}$	3.60 dB	9.10 dB		6.73 dB
R=2	50.9%	23.1%	26.0%	average
$\log\frac{E_{b}}{N_{0}}$	3.53 dB	8.15 dB	10.9 dB	7.68 dB

**Table 2:** Optimal power profile for the approximate upper bound, K=100 information bits per device, and per device block error rate $10^{-3}$.

R=0.4	100%						average
$\log\frac{E_{b}}{N_{0}}$	0.30 dB						0.30 dB
R=0.6	68.3%	31.7%					average
$\log\frac{E_{b}}{N_{0}}$	0.23 dB	1.73 dB					0.76 dB
R=0.8	52.8%	22.3%	24.9%				average
$\log\frac{E_{b}}{N_{0}}$	0.19 dB	1.86 dB	2.81 dB				1.36 dB
R=1	41.7%	23.3%	10.6%	24.2%			average
$\log\frac{E_{b}}{N_{0}}$	0.13 dB	1.93 dB	2.87 dB	3.92 dB			2.04 dB
R=1.2	34.5%	22.0%	3.16%	7.61%	11.3%	21.4%	average
$\log\frac{E_{b}}{N_{0}}$	0.10 dB	1.96 dB	3.24 dB	3.36 dB	3.74 dB	5.11 dB	2.76 dB

## Data Availability

Not applicable.

## Appendix A. Unconditional Block Error Probability

Let $z\in R^{NM}$ denote the vector of interference and noise. The ensemble-averaged block error probability for any device in group $G_{j}$ given the Euclidean norms of receive word r and interference-and-noise vector z, r=||r|| and z=||z||, respectively, is given by [11]

(A1) $$p_{j|r,z}=1-Q_{\frac{MN}{2}}{\left(\frac{r}{\sqrt{P_{j}/M}},\frac{z}{\sqrt{P_{j}/M}}\right)}^{2^{K}-1}$$

with $Q_{a}\left(b,c\right)$ denoting the generalized Marcum Q-function. Although the Euclidean norms of received word and interference-and-noise vector are not independent of each other, they can be constructed out of three statistically independent random variables χ, ζ, and γ [11] by

(A2) $$\begin{array}{cc} z^{2} & =\chi^{2}+\zeta^{2} \end{array}$$

(A3) $$\begin{array}{cc} r^{2} & =\chi^{2}+{\left(\zeta +\gamma \right)}^{2}. \end{array}$$

As discussed in [11], ζ and |χ| are the radial component and the Euclidean norm of the tangential component both of noise and interference, respectively. Furthermore, |γ| is the Euclidean norm of the transmitted codeword. Thus, ζ is zero mean Gaussian with variance *I*, $\gamma^{2}M/P_{j}$ and $\chi^{2}/I$ are chi-square distributed with MN and MN−1 degrees of freedom, respectively.

The conditional error probability can be written as

(A4) $$p_{j|\chi ,\zeta ,\gamma }=1-Q_{\frac{MN}{2}}{\left(\sqrt{\frac{\chi^{2}+{\left(\zeta +\gamma \right)}^{2}}{P_{j}/M}},\sqrt{\frac{\chi^{2}+\zeta^{2}}{P_{j}/M}}\right)}^{2^{K}-1}.$$

Both arguments of the generalized Marcum Q-function in (A4) linearly scale with *M*. The term $(\chi^{2}+\zeta^{2})/I$ is chi-square distributed with MN degrees of freedom. Its mean and standard deviation are MN and $\sqrt{2MN}$, respectively. Its distribution, if normalized by *M*, converges to a mass point at *N*. Due to the term $P_{j}/M$ in the denominator, the second argument of the generalized Marcum Q-function asymptotically scales linearly in *M*. The first argument is even slightly larger due to the addition of γ. However, γ does not scale with the number of devices, so asymptotically both terms scale in the same way. Thus, we are interested in the behavior of the generalized Marcum Q-function when all arguments grow over all bounds. In Appendix B, we show

(A5) $$\lim_{M^{\prime }\to \infty }Q_{aM^{\prime }}\left(M^{\prime }-\epsilon ,M^{\prime }\right)=Q\left(\epsilon -a\right)$$

with Q(·) denoting the standard Gaussian Q-function. Thus, we obtain

(A6) $$\begin{array}{cc} p_{j|\chi ,\zeta ,\gamma }\; & \overset{\cdot }{=}1-Q{\left(\sqrt{\frac{\chi^{2}+\zeta^{2}}{P_{j}/M}}-\sqrt{\frac{\chi^{2}+{\left(\zeta +\gamma \right)}^{2}}{P_{j}/M}}-\frac{N\sqrt{MP_{j}}}{2\sqrt{\chi^{2}+\zeta^{2}}}\right)}^{2^{K}-1} \end{array}$$

with $\overset{\cdot }{=}$ denoting asymptotic equivalence for M→∞. With probability approaching 1 for large *M*, we have

(A7) $$\chi^{2}\gg \zeta^{2}\;\wedge \;\chi^{2}\gg {\left(\zeta +\gamma \right)}^{2}.$$

Thus, we can develop the roots in (A6) into first order Taylor series at $\chi^{2}$ and obtain

(A8) $$\begin{array}{cc} p_{j|\chi ,\zeta ,\gamma }\; & \overset{\cdot }{=}1-Q{\left(\frac{-NP_{j}-\gamma^{2}-2\left|\gamma \right|\zeta }{2|\chi |\sqrt{P_{j}/M}}\right)}^{2^{K}-1} \end{array}$$

The random variable $\left|\gamma \right|\sqrt{M/P_{j}}$ is chi-distributed. Thus, its variance is upper bounded by $\frac{1}{2}$. This implies that the variance of |γ| vanishes for large *M*. This is in contrast to $\gamma^{2}$ and ζ which have variance $2NP_{j}$ and *I* given in (10), respectively. For M→∞, |γ| is arbitrarily closely approximated by its asymptotic mean $\sqrt{NP_{j}}$. Similar considerations imply that |χ| may be replaced by its asymptotic mean $\sqrt{IMN}$. This gives

(A9) $$\begin{array}{cc} p_{j|\chi ,\zeta ,\gamma } & \overset{\cdot }{=}p_{j|\zeta ,\gamma }\overset{\cdot }{=}1-Q{\left(\frac{-NP_{j}-\gamma^{2}-2\sqrt{NP_{j}}\zeta }{2\sqrt{INP_{j}}}\right)}^{2^{K}-1} \end{array}$$

The argument of the Q-function is the sum of a constant and two random variables with asymptotic distributions

(A10) $$\begin{array}{cc} -\frac{\zeta }{\sqrt{I}} & ∼N(0,1) \end{array}$$

(A11) $$\begin{array}{cc} -\frac{\gamma^{2}}{2\sqrt{INP_{j}}} & ∼N\left(-\frac{\sqrt{NP_{j}}}{2\sqrt{I}},\frac{1}{2IM}\right). \end{array}$$

The second random variable turns into a constant as M→∞. This implies

(A12) $$\begin{array}{cc} p_{j|\zeta ,\gamma } & \overset{\cdot }{=}p_{j|\zeta }\overset{\cdot }{=}1-Q{\left(\frac{-\sqrt{NP_{j}}-\zeta }{\sqrt{I}}\right)}^{2^{K}-1}. \end{array}$$

From the three random variables χ, ζ, and γ, only ζ has survived the infinite device limit. The variance of γ has vanished. The variance of χ has not vanished, but the influence of χ on the conditional block error probability has done so. It can be seen from [11] that ζ is the radial component of noise and interference relative to the true codeword. Averaging over the Gaussian random variable ζ, we obtain (11).

## Appendix B. Limit of the Generalized Marcum Q-Function

The noncentral chi-square distribution with *k* degrees of freedom and non-centrality parameter λ follows the CDF

(A13) $$1-Q_{\frac{k}{2}}\left(\sqrt{\lambda },\sqrt{x}\right)\to 1-Q\left(\frac{x-\mu }{\sigma }\right)$$

which converges to the Gaussian distribution of same mean μ and variance $\sigma^{2}$ due to the central limit theorem. We have

(A14) $$\begin{array}{cc} \mu & =k+\lambda ,\;\sigma^{2}=2k+4\lambda \end{array}$$

Letting k=2aM, $\lambda ={\left(M-\epsilon \right)}^{2}$, and $x=M^{2}$, we get

(A15) $$\begin{array}{cc} Q_{aM}\left(M-\epsilon ,M\right) & \to Q\left(\frac{x-k-\lambda }{\sqrt{2k+4\lambda }}\right)=Q\left(\frac{M^{2}-2aM-{\left(M-\epsilon \right)}^{2}}{\sqrt{4aM+4{\left(M-\epsilon \right)}^{2}}}\right) \end{array}$$

which for M→∞ converges to (A5).
